# COVID-19 in Africa: Survey Analysis of Impact on Health-Care Workers

**DOI:** 10.4269/ajtmh.20-1478

**Published:** 2021-04-22

**Authors:** Nasreen S. Quadri, Amir Sultan, Sophia Ibrahim Ali, Mirghani Yousif, Abdelmajeed Moussa, Ehab Fawzy Abdo, Sahar Hassany, Johnstone Kayandabila, Allison Benjamin, Mark Jacobson, Kenneth Ssebambulidde, Lucy Ochola, Ifeorah Ijeoma, Jose D. Debes

**Affiliations:** 1 University of Minnesota, Department of Medicine and School of Public Health, Minneapolis, Minnesota;; 2 Addis Ababa University, Department of Gastroenterology, Addis Ababa, Ethiopia;; 3 University of Gezira, School of Pharmacy, Gezira, Sudan;; 4 Aswan University Hospital, Department of Gastroenterology, Aswan, Egypt;; 5 Al-Rajhi University Liver Hospital–Assiut University, Department of Tropical Medicine and Gastroenterology, Assiut, Egypt;; 6 Arusha Lutheran Medical Centre, Department of Medicine, Arusha, Tanzania;; 7 Makerere University, Department of Medicine, Kampala, Uganda;; 8 Institute for Primate Research, Nairobi, Kenya;; 9 University of Nigeria, Department of Virology, Nsukka, Nigeria;; 10 Hennepin Healthcare, Minneapolis, Minnesota

## Abstract

As coronavirus disease 2019 (COVID-19) spreads across Africa, little is known about the impact of the pandemic on health-care workers (HCWs) in the region. We designed an anonymous survey distributed via e-mail and phone messaging to 13 countries through the African Hepatitis B Network. We obtained 489 analyzable responses. We used risk ratio analysis to quantify the relationship between binary variables and χ^2^ testing to quantify the statistical significance of these relationships. Median age of respondents was 30 years (interquartile range, 26–36 years) and 63% were physicians. The top three sources of information used by HCWs for COVID-19 management included the Ministry of Health of each country, the WHO, and social media. Forty-nine percent reported a decrease in income since the start of the pandemic, with the majority experiencing between a 1% and a 25% salary reduction. Sixty-six percent reported some access to personal protective equipment; only 14% reported appropriate access. Moreover, one third of respondents reported no availability of ventilators at their facility. Strikingly, the percentage of HCWs reporting never feeling depressed changed from 61% before the pandemic to 31% during the pandemic, with a corresponding increase in daily depressive symptoms from 2% to 20%. Most respondents (> 97%) correctly answered survey questions about COVID-19 symptoms, virus transmission, and prevention. Our survey revealed African HCWs face a variety of personal and professional context-dependent challenges. Ongoing support of HCWs through and after the COVID-19 pandemic is essential.

## INTRODUCTION

As the novel severe acute respiratory syndrome coronavirus 2 (SARS-CoV-2) virus is spreading universally across the world, the impact of coronavirus disease 2019 (COVID-19) (the disease caused by SARS-CoV-2) on health-care workers (HCWs) and health-care systems is determined largely by geography and subsequent availability of resources. This is becoming increasingly evident in Africa.^1^ Disproportionate effects of COVID-19 across the African continent are rooted heterogeneously in unique context-dependent challenges. Rosenthal et al.^2^ identify priority areas for COVID-19 containment in Africa, including community ownership and action, implementation of low-cost sanitary measures, risk communication and disease prevention, as well as addressing economic impact. A study analyzing demographic and health survey results from 16 sub-Saharan African countries found on average only 33.5% of households with an observed handwashing place had water and soap access, with notable differences based on country, urban versus rural location, and wealth quintiles.^3^ In addition, high-density living conditions make social distancing less feasible. Median household size across sub-Saharan Africa was found to be 4.8 individuals based on results from the 2019 United Nations Database on Household Size and Composition.^4^

As the world collectively discovered more about the novel SARS-CoV-2 virus, African countries were tasked with finding mitigation strategies to work in a local context. Early during the pandemic, several strategies were used to optimize early response efforts in Africa: the African Union established a taskforce for Coronavirus preparedness, and resources from other diseases such as Ebola were re-allocated toward COVID-19.^5^ Each country mobilized around unique actions: South Africa deployed 30,000 HCWs for screening efforts, the Democratic Republic of the Congo and Liberia mobilized Ebola resources for contact tracing, and Rwanda alongside eight other Sub-Saharan African countries swiftly enacted travel restrictions and country lockdown at the onset of the pandemic.^5^ Regional responses were notably swift and strong within the global context, but the potential for the burden of COVID-19 cases overwhelming medical capacity remains if complacency is adopted similar to any other global context.^6^ Global HCW shortage is demonstrably apparent by the stark differences in ratios of HCWs per population. Africa has 2.3 HCWs per 1,000 population, compared with the Americas, which have 24.8 HCWs per 1,000 population.^7^ Nurses alone comprise half of the global healthcare workforce, with shortages of nurses disproportionately impacting regions such as Africa.^8^ This is compounded by the pre-existing medical system scarcity in the aftermath of colonialism and years of resource extraction.^9^ The impact of the COVID-19 pandemic on HCWs remains mystified because it is largely dependent on country-specific policies, individual resource access, and burden of disease in the specific country.

Although multiple studies have aimed to understand the impact of the pandemic on individuals and institutions, few have focused on the impact on HCWs. Understanding perceptions and realities affecting the health-care community in Africa will allow for identification of potential targets for prevention and mitigation of burnout. With this in mind, we developed and distributed a pan-African survey to assess the impact of the COVID-19 pandemic on HCWs in the continent.

## METHODS

### Survey.

We designed a 43-question survey that was divided into four sections (demographics, personal, work, COVID-19) composed mainly of multiple-choice answers, with a component of free-style answers sections related to social discrimination. The demographics subsection inquired about basic information such as age, gender, profession, country, city, and whether there was a formal lockdown implemented in the region. The personal subsection inquired about household size, transportation utilization, safety concerns, depression, alcohol use, religious services, social discrimination, and fears. The work subsection inquired about access to personal protective equipment (PPE), ventilator availability, changes in workload or income, and institution preparedness for COVID-19, including isolation beds and resources to access the latest medical recommendations. The last section surveyed HCW knowledge about COVID-19, including treatment, prevention, knowledge, and misinformation, and gathered sources of reliable information used for evolving COVID-19 management. A full description of the survey is available in Supplemental materials.

### Study population.

The survey was anonymous and distributed via e-mail and phone messaging to individuals in 13 countries through the African Hepatitis B Network (africanhepbnetwork.org). A mock survey was initially distributed among five partners from unique institutions in five different countries in Africa for feedback on clarity and feasibility. Later, the survey was distributed to members of the African Hepatitis B Network, who subsequently shared the survey in different HCW forums across their respective countries, resulting in snowball sampling. The African Hepatitis B Network, which was founded in 2015, currently has 14 members (doctors, nurses, and pharmacists) from 12 different countries. The first-seed round distribution through the African Hepatitis B Network was sent to 17 individuals: three people in Tanzania from three different institutions, two people in Uganda representing unique institutions, two people in Nigeria affiliated with two different institutions, one person in Kenya, two people in Sudan representing two institutions, one person in Somalia, two people in Ethiopia from unique institutions, one person from Sierra Leone, and three people from Egypt representing three different institutions. The definition of snowball sampling used refers to chain referral sampling in which the first seed of distribution shared the survey with contacts within their networks, although not limited only to their affiliated institutions or their own country. The ethical clearance of the study was submitted to the Hennepin Healthcare ethics committee and was approved by the Hennepin Healthcare Institutional Review Board. The survey was distributed in April and May 2020.

### Analysis.

Responses were integrated into an Excel Version 16.43 (Microsoft Corp., Redmond, WA) platform for visualization and interpretation of results. We received a total of 535 responses between April 22, 2020 and May 15, 2020. After excluding duplicate submissions and incomplete responses (those with answers to fewer than four of the survey questions), 489 responses were used for analysis. We used RR analysis to quantify the relationship between binary variables and χ^2^ testing to quantify the statistical significance of these relationships. Multiple-choice survey responses were converted into binary variables for relative risk analysis by 2 × 2 tables when applicable. Household size was converted into binary variables of ≤ 4 or > 4, using median household size of four as a cutoff point. The Patient Health Questionnaire-2 (PHQ-2) was used as a framework to screen for depression by asking survey respondents how often they felt down, depressed, or hopeless both before the pandemic began and during the current pandemic.^10^ Self-reported depression preceding and since the pandemic was recoded into “yes” (those who reported any level of depression) and “no” (those who reported never to be depressed). The PHQ-9 and PHQ-2 have been validated with reasonable accuracy to classify depression in Sub-Saharan Africa.^11–14^ Free-form responses for survey questions inquiring about HCW fears for personal safety were recoded into yes or no. Change in work or school frequency was recoded into binary variables for which yes correlated to more or less frequency and no correlated to the same frequency. Access to PPE was recoded into binary variables for which yes correlated to any access to PPE and no correlated to no access to PPE. Relative risk analysis was computed through RRs between self-reported depression since the pandemic began and selected survey questions (household size, enforced lockdown/stay-at-home recommendation, changes in school/work frequency, decrease in income, and access to PPE). Analysis of relative risk was also applied to determine any relationship between profession type or access to PPE and suspected self-exposure to SARS-CoV-2. RRs were used to correlate both personal safety fears and concerns for exposing their families to the virus, with variables of household size > 4 and access to PPE. Last, a relative risk analysis was conducted to determine the correlation between a decrease in non-COVID-19 patients to their work facilities and a decrease in income.

## RESULTS

### Demographics.

**Figure 1.**
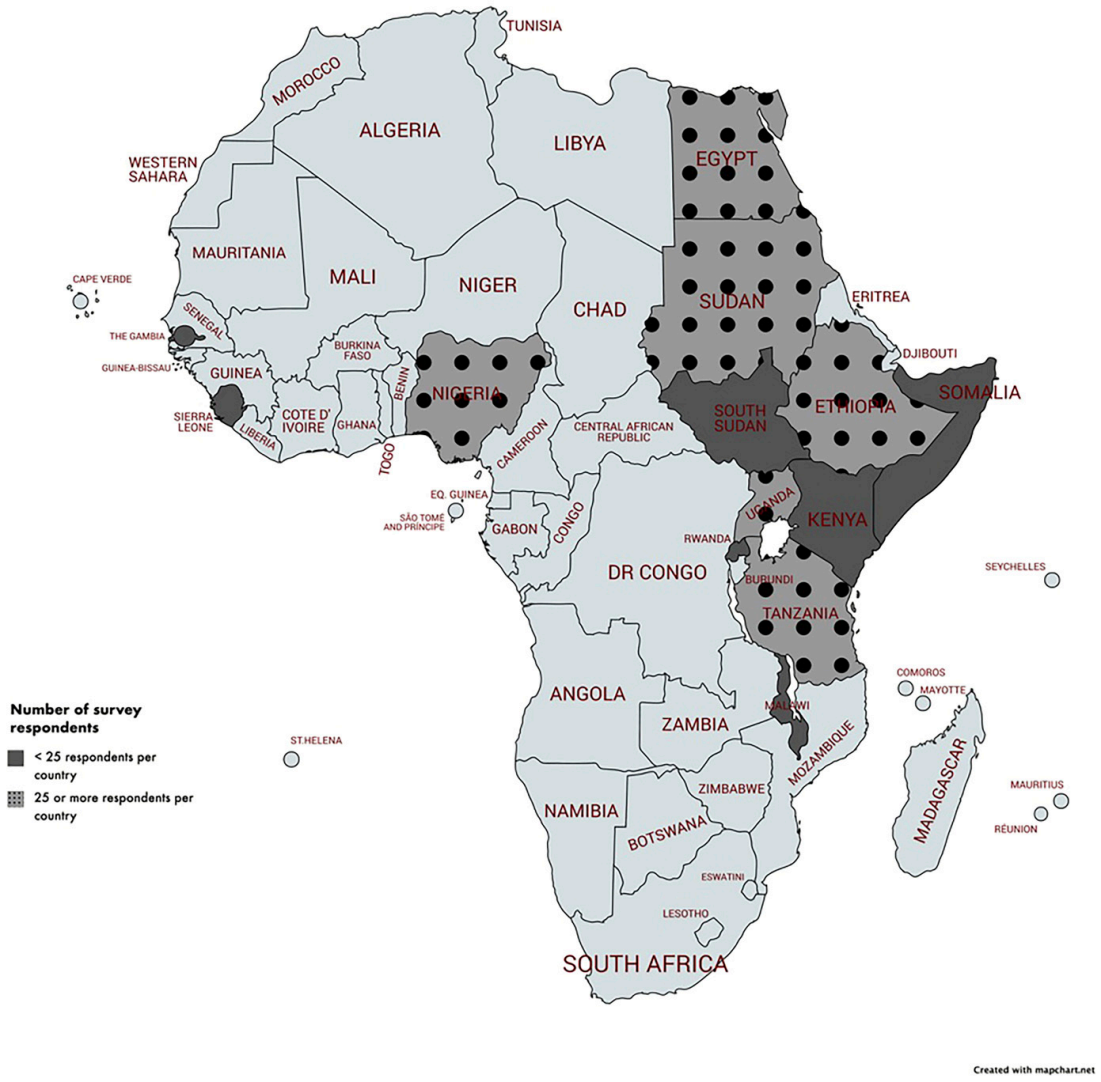
Country of residence for survey respondents. Countries in dark gray represent those from which surveys were received. Countries in light gray with black dots represent those from which 25 or more survey responses were received. This figure appears in color at www.ajtmh.org.

A total of 13 countries across Africa were represented among the final 489 analyzable responses to the survey. Respondents from six countries (Nigeria, Ethiopia, Sudan, Uganda, Tanzania, and Egypt) provided at least 25 responses per country (Figure 1). The remaining respondents were from Kenya, Sierra Leone, Somalia, The Gambia, Rwanda, South Sudan, and Malawi. Ethiopian HCWs provided 243 responses (51%) alone. The median age of respondents was 30 years (interquartile range, 26–36 years), 73% identified as male, and 62% as physicians. Of note, 18% of physicians were consultants and the remaining 44% were trainee physicians, either an intern, registrar, or resident. The professions of the remaining respondents included pharmacists, nurses, students, or clinical/medical officers. Demographic data are summarized in Table 1.

**Table 1:** Demographics of survey respondents

Country	Profession type, *N* (%)							Gender, *N* (%)	
	Doctor: consultant	Doctor: intern, registrar, resident	Medical officer, clinical officer	Nurse	Pharmacist	Student	Other	Female	Male
Egypt	19 (56)	15 (44)	–	–	–	–	–	14 (41)	20 (59)
Ethiopia	22 (9)	157 (65)	12 (5)	12 (5)	6 (3)	24 (10)	7 (3)	49 (20)	192 (80)
Kenya	3 (13)	1 (4)	1 (4)	1 (4)	4 (17)	–	14 (58)	11 (46)	13 (54)
Nigeria	4 (10)	1 (2)	8 (20)	5 (12)	1 (2)	–	22 (54)	11 (25)	33 (75)
Somalia	0 (0)	1 (11)	3 (33)	5 (56)	–	–	–	1 (11)	8 (89)
Sudan	12 (43)	1 (4)	–	–	13 (46)	–	2 (7)	11 (39)	17 (61)
Tanzania	22 (34)	26 (41)	5 (8)	3 (5)	–	3 (5)	5 (8)	22 (35)	41 (65)
Uganda	2 (8)	4 (16)	10 (40)	3 (12)	2 (8)	1 (4)	3 (12)	7 (28)	18 (72)
Malawi	1 (50)	–	–	–	–	–	1 (50)	1 (50)	1 (50)
Sierra Leone	–	–	–	2 (100)	–	–	–	2 (100)	–
Rwanda	–	–	–	1 (100)	–	–	–	–	1 (100)
South Sudan	–	1 (100)	–	–	–	–	–	–	1 (100)
The Gambia	–	–	–	–	–	–	1 (100)	–	1 (100)
Total	85 (18)	207 (44)	39 (8)	32 (7)	26 (6)	28 (6)	55 (12)	129 (27)	346 (73)

### Household and transportation.

The median household size for HCWs in our survey was four individuals, whereas 27% of respondents had a household size of six or more people. A notable 89% of respondents indicated worry about exposing their family to SARS-CoV-2 because of the high-risk nature of their work. Concerns about family exposure to the virus did not correlate significantly with household size > 4 (RR, 0.99; CI, 0.93–1.06; *P* = 0.86) or access to PPE (RR, 1.05; CI, 0.96–1.14; *P* = 0.24). Survey results revealed 72% of respondents living under stay-at-home orders, with some intercountry variation, and a smaller subset (16%) under enforced lockdown orders. These population level mandates affected our respondents’ work schedules and modes of transportation to access their work. Since the beginning of the pandemic, 54% of HCWs reported going to work less frequently, and 15% more frequently than usual. The majority of respondents (51.7%) reported traveling to work by car, followed by walking (41.5%), with fewer traveling by bus (25%) or bicycle (5.4%). Transportation patterns were similar prior to the pandemic, although there was previously greater use of travel by bus (31.9%) and a lower dependence on walking (35.9%).

### Institutional preparedness and availability of PPE.

Facility preparedness inquiries revealed that 64% of HCWs confirmed specific isolation rooms allocated for COVID-19 patients at their institutions. The majority of respondents (73%) reported decreased volume of non-COVID-19 patients to their facility. In the absence of in-person visits, 41% had access to alternate platforms for providing care, such as video calls or phone calls. Sixty-six percent reported some access to PPE, 20% had no access to PPE, and only 14% had proper access. Most respondents had access to gloves (81.8%) and surgical masks (82.2%), with less availability of gowns (36.2%) and N95 respirator masks (24.3%). Powered air-purifying respirators/controlled air-purifying respirators were available only for 1.6% of respondents. Only 42% of survey respondents suspected personal specific exposure to COVID-19 at the time of the survey distribution. Physicians were more likely to report suspected exposure to the virus than individuals in other professions (e.g., nurses, students, clinical officers/medical officers, pharmacists), with an RR of 1.82 (CI, 1.41–2.37, *P* < 0.0001). This correlation was not found in nurses and suspected exposure (RR, 0.65; CI, 0.37–1.15; *P* = 0.10). Surprisingly, there was no statistically significant relationship between suspected SARS-CoV-2 exposure and access to PPE (RR, 0.81; CI, 0.64–1.03; *P* = 0.10). In addition, only 25% personally knew a HCW in their area that became ill from SARS-CoV-2 during the time frame of the survey distribution. Nearly 29% of our respondents reported their facility had no functional ventilators, the majority (31.2%) had between one and five ventilators, and only 13.7% of respondents reported facility access to > 10 functional ventilators (Table 2). A marked 49% of respondents had a decrease in income, with the majority experiencing a 1 to 25% salary reduction (Table 3). Interestingly, the RR comparing the variables of decreased income with a decrease in non-COVID-19 patients to their facility was not statistically significant (RR, 0.88; 95% CI, 0.69–1.12; *P* = 0.32).

**Table 2:** Ventilator access

No. of ventilators	No. of respondents	Percentage of respondents
> 10	65	14
5–10	51	11
1–5	144	31
None	133	28
I don’t know	74	16
Total	467	100

**Table 3:** Income changes since the pandemic

Percentage change in income	No. of respondents	Percentage of respondents
> 50% less income	36	10
25–50% less income	65	18
1–25% less income	74	21
No change to income	182	51
Total	357	100

### Use of medications for COVID-19 and misinformation.

Most respondents (69%) reported facility guidance on consultation materials for management of COVID-19. The top three sources used by HCWs for COVID-19 information were the Ministry of Health of each country, the WHO, and social media. Interestingly, 13% reported treating COVID-19 with medications—mainly, hydroxychloroquine, chloroquine, and azithromycin. Of note, at the time of distribution of the survey, no medication had been approved in any country for the treatment of COVID-19. Other classes of medications survey respondents used for management included antipyretics, antivirals, vitamins, antibiotics, anticoagulants, and steroids. Tanzania, Egypt, and Ethiopia were the countries where the most respondents reported using medications for management of COVID-19. A set of knowledge-based questions were included as part of the survey. More than 97% of survey respondents correctly identified COVID-19 symptoms, transmission routes, risk factors for developing severe disease, and prevention measures. Approximately 70% reported ongoing misinformation in the community, including unproven cures, false modes of transmission, and speculated origin of the virus.

### COVID-19 and lifestyle impact.

**Figure 2.**
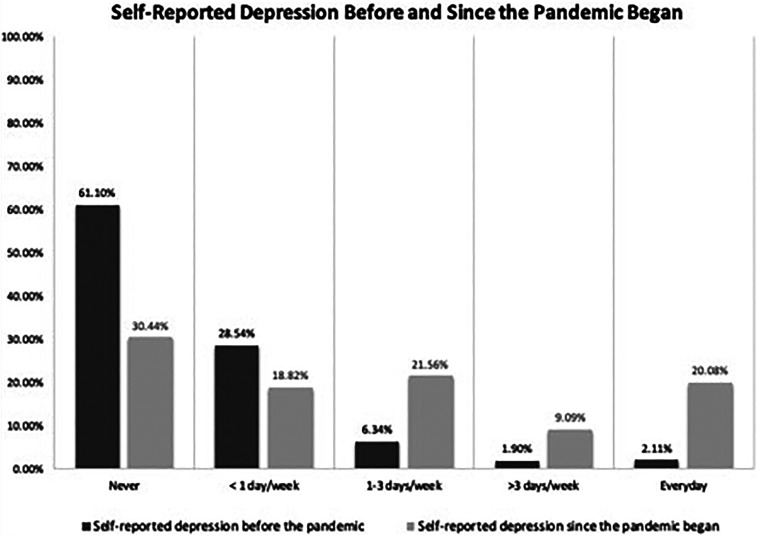
Self-reported depression before and since the pandemic began. The *y* axis represents the percentage of responses to the specific question; the *x* axis represents the frequency of reported depression. Darker bars represent before the pandemic; lighter bars represent since the start of the pandemic.

Beyond the professional impact to their spheres of work, HCWs noted the impact of the COVID-19 pandemic on their own health and well-being. The PHQ-2 was used as a framework to screen for depression by asking survey respondents how often they felt down, depressed, or hopeless both before the pandemic began and during the current pandemic.^10^ Strikingly, the percentage of HCWs reporting never feeling depressed changed from 61% before the pandemic to 31% during the pandemic, with a corresponding increase in daily depressive symptoms from 2 to 20% (Figure 2). There was a statistically significant inverse association between self-reported depression and any change in work frequency (RR, 0.86; CI, 0.76–0.96; *P* = 0.02). There was no statistically significant relationship between the variable of self-reported depression with specific selected survey questions: household size > 4 (RR, 1.10; CI, 0.89–1.14; *P* = 0.09), lockdown recommendation (RR, 0.94; CI, 0.82–1.07; *P* = 0.34), decreased income (RR, 1.08; CI, 0.96–1.22; *P* = 0.23), access to PPE (RR, 0.95; CI, 0.82–1.11; *P* = 0.55), and allocation of COVID-19 isolation rooms at their facility (RR, 0.95; CI, 0.84–1.08; *P* = 0.43) (Table 4). We found a nonsignificant increase of alcohol consumption; 74% reported never drinking before the pandemic and 68% during the pandemic, with no change in rates of daily drinking (< 1% both preceding and since the pandemic began). Of respondents that attend a religious service regularly, 78% confirmed cancellation of in-person religious services. Most HCWs reported access to alternative forms of connecting to religious services including TV (48%), social media (39%), and radio (13%).

**Table 4:** Relative risk ratio analysis

Variable	Risk ratio (CI)	*P* value
HCW concerns about exposing family to SARS-CoV-2		
Household size > 4	0.99 (0.93–1.06)	0.86
Access to PPE	1.05 (0.96–1.14)	0.24
HCW-suspected personal exposure to SARS-CoV-2		
Profession, doctor	1.82 (1.41–2.37)	< 0.0001
Profession, nurse	0.65 (0.37–1.15)	0.10
Access to PPE	0.81 (0.64–1.03)	0.10
HCW-reported decreased incomed since pandemic		
Decrease in non-COVID-19 patients	0.88 (0.69–1.12)	0.32
HCW self-reported depression or hopelessness		
Household size > 4	1.01 (0.89–1.14)	0.89
Lockdown or stay-at-home recommendation	0.94 (0.82–1.07)	0.34
Change in work/school attendance frequency	0.86 (0.76–0.96)	0.02
Decrease in income	1.08 (0.96–1.22)	0.23
Access to PPE	0.95 (0.82–1.11)	0.55
HCW self-reported fear for personal safety		
Household size > 4	1.16 (1.01–1.34)	0.04
Access to PPE	0.85 (0.73–1.00)	0.07

COVID-19 = coronavirus disease 2019; HCW = health-care worker; PPE = personal protective equipment; SARS-CoV-2 = severe acute respiratory syndrome coronavirus 2.

Fifty-six percent of the surveyed HCWs attested to safety concerns regarding COVID-19. Fears centered on risk of infection resulting from lack of resources (33%), risk of infection as a result of community transmission (23%), economic insecurity (11%), governmental concerns (10%), and social stigma (11%). The risk for personal safety correlated positively with household size > 4 in a statistically significant way (RR, 1.16; CI, 1.01–1.34; *P* = 0.04), although it was not correlated significantly with access to PPE (RR, 0.85; CI, 0.73–1.00; *P* = 0.07).

## DISCUSSION

Our survey responses offer a snapshot of the varied impact of COVID-19 across Africa early during the course of the pandemic. The results of this survey provide important insights into the current state of COVID-19 and its uneven tread across the globe. Notably, lockdown measures were implemented globally to help limit the mobility and gathering of individuals to slow the spread of SARS-CoV-2. Mboera et al.^15^ define aspects of lockdown relevant to COVID-19 as “geographical containment”; “home confinement”; “the closure of social, educational, and economic activities”; and “the prohibition of mass gatherings.” Countries used a varying degree of implementation and further enforcement of these areas of restriction. In addition, countries across Africa were also burdened with balancing lockdown mandates with the undesired effects of impeding on civil liberties, disrupting education for children, reducing access to food, and impacting the financial security of its citizens negatively.^15^ Our survey results revealed that most countries used some form of restriction, although enforcement may have been region specific, given our results showed regional variability even within the same country. Consequently, participant responses suggested a dramatic increase in feelings of hopelessness and depression compared with before the pandemic began. Although partially expected, these changes are of concern and require attention from institutions to support HCWs further in this critical factor of provider burden.

There is much to be deciphered about the work environment for HCWs across Africa in the time of COVID-19. The results of our survey revealed that the impact of the virus was associated with change in workflow and income, access to PPE and resources, and societal shifts with the aforementioned lockdown mandates. Ventilator availability has been highlighted globally as a concern regarding resource scarcity. Ventilator access in the African context is not only limited to equipment availability, but factors to consider include power supply and oxygen availability in addition to trained personnel with adequate familiarity and experience to use the equipment. Madzimbamuto^16^ astutely concluded that focusing efforts on obtaining oxygen supplies, concentrators, and oximeters represents a more viable strategy for Africa than focusing on ventilators, because these supplies can be used more efficiently in the context of power outages and the reality of staff capabilities, and offer the opportunity for ongoing use after the pandemic. Our survey results confirm the established relative scarcity of ventilators in African facilities, making further emphasis on the need to focus on other noninvasive ventilation–oxygenation methods.

HCWs expressed fears for the safety of their families because they are at high risk of harboring and transmitting the novel SARS-CoV-2 to their household contacts. Ongoing fears were related to the stigma of being an HCW, overcrowding, and the economic impact of the virus on their communities. Our survey results were in concordance with U.S. findings of depression symptom prevalence 3-fold greater during the pandemic compared with before the pandemic.^17^ Our survey respondents similarly reported increased prevalence of depression symptoms during the pandemic than before. In contrast, alcohol consumption patterns reported in our survey differed from changes reported in resource-rich countries, reflecting increases in alcohol intake during the pandemic related to income loss and solitary living imposed by physical distancing.^18^ Levels of religious commitment are among the greatest globally in Sub-Saharan Africa based on the Pew Research Center^19^ analysis of data from 2008 to 2017. Religious services are a common part of cultural practice across the continent, and the current pandemic has affected how faith organizations have offered their services. Interestingly, our survey found that many faith organizations adapted to the pandemic circumstances and offered alternate forms of religious services using radio, TV, and social media to reach the community.

Limitations of our survey include use of the English language only, online distribution, and the snowball sampling method. This may have excluded those for whom English is not their preferred language, those with challenges in Internet connectivity, and those not integrally connected to our network of colleagues in Africa through the African Hepatitis B Network. There was a risk of availability bias for questions regarding depression and alcohol use before the pandemic and currently at the time of survey distribution. Survey instructions explicitly gave respondents the option to leave the answers to a question blank if they did not feel comfortable sharing that information. This resulted in a smaller sample size of certain survey answers and potential for underrepresentation of all survey perspectives for each question. However, the analysis of each question and the statistical analysis were not affected negatively by this limitation. The limitation to RR analysis through dichotomizing multicategory survey responses into binary categories has the potential to mask the variability of responses within the two categories. Because this was intended to be a descriptive study completed with snowball sampling with no prespecified hypotheses, we did not complete a formal power or sample size analysis a priori. As a result, we may not have adequate power to achieve statistical significance for meaningful associations in some cases. Although, we note that our sample size was large enough to detect statistically significant differences for moderate to large effect sizes in many cases. Regarding institutional bias limiting generalizability, closer review of our data reveals that the survey was completed by individuals from 214 unique institutions. Last, guidance and practice have changed as the COVID-19 pandemic evolved, and thus a follow-up survey for respondents could provide further context to current conditions.

In summary, our survey revealed dramatic personal and professional effects of the COVID-19 pandemic on HCWs in Africa, with concerns of social stigma, an increase in self-reported depression, and concerns for personal safety. Local institutions and governments across the African continent should provide support for HCWs because these effects will impact their ability to provide needed care during the pandemic both related to COVID-19 and to other diseases. Moreover, HCW well-being is critical for the post-pandemic period to ensure a rapid recovery, with an emphasis on effective and proper care of the population.

## Supplemental material

Supplemental materials
