# Supplementary material for: COVID-19 in Africa: Survey Analysis of Impact on Health-Care Workers
Source: Am J Trop Med Hyg. 2021 Apr 22;104(6):2169–75. doi: 10.4269/ajtmh.20-1478 (PMC8176463; doi:10.4269/ajtmh.20-1478)
Supplement: Supplementary file 1 [file tpmd201478.SD1.pdf]

African Context of COVID-19

We are conducting a survey to understand how the COVID-19 pandemic is affecting healthcare workers across Africa. This survey is coordinated by researchers at the University of Minnesota. Your response will help us in identifying issues that might be common across countries (the survey is conducted in over 12 African countries). The survey is totally anonymous and there is no link to personal information. Please answer questions honestly to the best of your ability. If you feel uncomfortable at any time, you may leave the answer to a question blank. Thank you for your help with this survey!

DEMOGRAPHIC INQUIRIES

1. What is your gender?

Mark only one oval.

- Female
- Male
- Prefer not to say

2. What is your age?

____

3. What is your profession?

Mark only one oval.

- Doctor - Consultant
- Doctor - Intern/Registrar/Resident
- Nurse
- Medical Officer or Clinical Officer or similar
- Pharmacist
- Student
- Other

4. Have you been in your work or school position for over 1 year?

Mark only one oval.

- Yes
- No

5. Which country do you live in?

____

6. Which city do you live in?

____

7. What is the name of the facility where you work? (This information will not be made public and will remain anonymous. It will be used to understand the incidence of COVID-19 in your area).

____

8. Does your city recommend or enforce any form of lockdown?

Mark only one oval.

- ( ) Yes
- ( ) No

PERSONAL INQUIRIES

9. How many people live in your house (including you)?

____

10. How do you travel to work/school? More than one box may be checked.

Check all that apply.

- [ ] Bus
- [ ] Car
- [ ] Walk
- [ ] Bike
- [ ] Other

11. How did you travel to work/school before the pandemic? More than one box may be checked.

Check all that apply.

- [ ] Bus
- [ ] Car
- [ ] Walk
- [ ] Bike
- [ ] Other

12. Do you have concerns about your personal safety due to COVID-19 lockdown in your country? If yes, then why? If no, please write no.

13. How often have you been feeling down, depressed or hopeless since the COVID-19 pandemic began?

Mark only one oval.

- ( ) Never
- ( ) < 1 day/week
- ( ) 1-3 days/week
- ( ) >3 days/week
- ( ) Everyday

14. If you feel down, depressed or hopeless, please state why? More than one box may be checked.

Check all that apply.

- [ ] Due to being overworked currently
- [ ] Due to being in lockdown at home
- [ ] Due to fear of illness and/or death from COVID-19
- [ ] Due to social discrimination as a result of being a healthcare provider
- [ ] I do NOT feel down, depressed or hopeless

15. How often did you feel down, depressed or hopeless last year before the pandemic?

Mark only one oval.

- Never
- <1 day/week
- 1-3 days/week
- >3 days/week
- Everyday

16. Do you drink alcohol? If so, how many drinks (one glass)?

Mark only one oval.

- None
- <1 drink/week
- 1-3 drinks/week
- 3-5 drinks/week
- Daily

17. How much alcohol did you drink last year before the pandemic?

Mark only one oval.

- None
- <1 drink/week
- 1-3 drinks/week
- 3-5 drinks/week
- Daily

18. If you attend religious services, have in-person services been cancelled?

Mark only one oval.

- Yes
- No
- I do NOT attend frequently

19. If your religious services have been cancelled, have alternative forms of connecting to religious services been provided? If so, which platforms? More than one box may be checked.

Check all that apply.

- [ ] I do NOT attend frequently
- [ ] In-person services were NOT cancelled
- [ ] TV
- [ ] Social media
- [ ] Radio
- [ ] Other

20. Have you experienced any social discrimination due to being in a healthcare job? If yes, explain how. More than one box may be checked.

Check all that apply.

- [ ] I've been asked to move to another house/neighborhood
- [ ] I've been asked not to go to the market/pharmacy
- [ ] I've been asked not to leave my house
- [ ] Other

21. Are you worried about exposing your family to the virus due to your high risk of work exposure?

Mark only one oval.

- ( ) Yes
- ( ) No

22. Do you think you have had a specific exposure to COVID-19?

Mark only one oval.

- Yes
- No

WORK

23. Do you have access to personal protective equipment (PPE) like gloves, masks and gowns at your facility?

Mark only one oval.

- Yes, I have adequate access to PPE
- Yes, but I have limited access to PPE
- No, I do NOT have access to PPE

24. Which type of personal protective equipment (PPE) is commonly used at your facility? More than one box may be checked.

Check all that apply.

- [ ] Gloves
- [ ] Surgical masks
- [ ] N95 masks
- [ ] Gowns
- [ ] PAPR/CAPR (Powered Air-Purifying Respirator)
- [ ] I do NOT have access to any of these PPE items

25. How many functional ventilators are available at your facility?

Mark only one oval.

- None
- 1-5
- 5-10
- >10
- I don't know

26. Does your facility have rooms specifically prepared for COVID-19 positive patients?

Mark only one oval.

- Yes
- No

27. Have you seen a decreased number of non-COVID-19 patients coming to your facility?

Mark only one oval.

- Yes
- No

28. Since the COVID-19 pandemic began, have you been going to work/school with any change in frequency?

Mark only one oval.

- More frequently
- Less frequently
- Same frequency

29. Has there been a decrease in your income due to the ongoing COVID-19 pandemic?

Mark only one oval.

- Yes
- No

30. If yes to the last question, how much has your income decreased? Ex. Monthly salary decrease from 100 to 80 would be a 20% decrease [100 minus 80 = 20; 20 divided by 100= 20%]

Mark only one oval.

- No change
- 1-25% less income
- 25-50% less income
- >50% less income

31. Where do you get most of your COVID-19 information from? More than one box may be checked.

Check all that apply.

- [ ] Word of mouth
- [ ] Phone messages (including apps like Telegram, WhatsApp, etc)
- [ ] News-TV
- [ ] Social Media
- [ ] WHO
- [ ] African CDC
- [ ] Ministry of Health in your country
- [ ] Medical Literature
- [ ] Guidelines from other countries
- [ ] Other

32. Does your hospital/clinic/school provide a source of information about COVID-19 to consult?

Mark only one oval.

- ( ) Yes
- ( ) No

33. Are you treating COVID-19 with medications? If yes, which specific medications? If no, please type no.

____

34. Have you heard misinformation about COVID-19 circulating in the community? What specifically have you heard?

35. Does your facility provide alternate forms of providing care for your patients (video calls, phone calls, other)?

Mark only one oval.

- Yes
- No

36. Do you know any healthcare worker in your area that has been ill from COVID-19? If yes, how many?

Mark only one oval.

- No
- Yes, 1 person
- Yes, 1-5 people
- Yes, > 5 people

COVID-19 Questions

37. What are frequent COVID-19 symptoms?

Mark only one oval.

- Fever, chest pain, double vision
- Fever, cough, shortness of breath, diarrhea
- Joint pain, vomiting, skin rash, dark urine

38. What is the average amount of time after an exposure for COVID-19 to induce symptoms in an affected person?

Mark only one oval.

- 1-2 days
- 3-6 days
- 7-21 days

39. How is the SARS-CoV-2 virus (COVID-19) transmitted? Select the best answer.

Mark only one oval.

- Stool
- Urine
- Respiratory Droplets and Aerosols
- Sexual transmission

40. Who is most at risk of developing severe disease with COVID-19?

Mark only one oval.

- People with increased alcohol intake, eczema, frequent ear infections
- People > 65 years of age, chronic renal disease, chronic lung disease
- Children < age 18, people with chronic anemia, people with vision deficits

41. Why are cloth masks for the public recommended?

Mark only one oval.

- They work as well as medical masks
- They protect the person wearing from getting sick from community exposure
- They protect the community from acquiring disease from the person wearing the mask

42. What is the average number of people who can get the virus from exposure to one positive case with COVID-19?

Mark only one oval.

- 1
- 2.5
- 5
- 10

43. Which of the following are appropriate measures to prevent the spread of SARS-CoV-2?

Mark only one oval.

- ◯ Washing hands frequently
- ◯ Keeping a distance of 1.5-2 meters away from other people
- ◯ Avoiding shaking hands with other people
- ◯ All of the above

This content is neither created nor endorsed by Google.

Google Forms
